# Periodontitis: A risk factor to respiratory diseases

**DOI:** 10.4103/0970-2113.68313

**Published:** 2010

**Authors:** Rajiv Saini, Santosh Saini, Sugandha Sharma

**Affiliations:** *Department of Periodontology and Oral Implantology, Rural Dental College-Loni, Maharashtra, India. E-mail: drperiodontist@yahoo.co.in*; 1 *Department of Microbiology, Rural Dental College-Loni, Maharashtra, India*; 2 *Department of Prosthodontics, Rural Dental College-Loni, Maharashtra, India*

Sir,

Periodontitis is a destructive inflammatory disease of the supporting tissues of the teevth and is caused by specific microorganisms or group of specific microorganisms resulting in progressive destruction of periodontal ligament and alveolar bone with periodontal pocket formation, gingival recession or both.[1,2] The host responds to the periodontal infections with an array of events involving both innate and adaptive immunity. Periodontitis has been proposed as having an etiological or modulating role in cardiovascular and cerebrovascular disease, diabetes, respiratory disease and adverse pregnancy outcome and several mechanisms have been proposed to explain or support such theories. Oral lesions are indicators of disease progression and oral cavity can be a window to overall health.[3] Bacteria are the prime etiological agents in periodontal disease, and it is estimated that more than 500 different bacterial species are capable of colonizing the adult mouth[1] and the lesions of the oral cavity have an immense impact on the quality of life of patient with complex advance diseases.[3] Respiratory infectious diseases such as bacterial pneumonia and bronchitis are common and costly, especially in institutionalized and elderly inpatients. Respiratory infection is thought to rely in part on the aspiration of oropharyngeal flora into the lower respiratory tract and failure of host defense mechanisms to eliminate the contaminating bacteria, which then multiply to cause infection. It has been suggested that dental plaque may act as a reservoir of respiratory pathogens, especially in patients with periodontal disease.[4] Several mechanisms have been proposed to explain the potential role of oral bacteria in the pathogenesis of respiratory infection, which include the following: (1) aspiration of oral pathogens (such as Porphyromonas gingivalis, Actinobacillus actinomycetemcomitans, etc.) into the lung to cause infection; (2) periodontal disease-associated enzymes in saliva may modify mucosal surfaces to promote adhesion and colonization by respiratory pathogens, which are then aspirated into the lung; (3) periodontal disease-associated enzymes may destroy salivary pellicles on pathogenic bacteria to hinder their clearance from the mucosal surface; and (4) cytokines originating from periodontal tissues may alter respiratory epithelium to promote infection by respiratory pathogens.[5] In elderly patients living in chronic care facilities, the colonization of dental plaque by pulmonary pathogens is frequent. Notably, the overreaction of the inflammatory process that leads to destruction of connective tissue is present in both periodontal disease and emphysema. This overreaction may explain the association between periodontal disease and chronic obstructive pulmonary disease, the fourth leading cause of death in the United States; these findings underline the necessity for improving oral hygiene among patients who are at risk and those living in long-term care institutions.[6] Thus, it is concluded that lesions of oral cavity have an immense impact on the quality of life of patient with complex advanced diseases.[3] Oral diseases especially periodontitis should be treated on priority basis to maintain the overall health and to minimize the risk for systemic infections and diseases.

## References

[1] Saini R, Marawar PP, Shete S, Saini S (2009). Periodontitis a true infection. J Global Infect Dis.

[2] D’Aiuto F, Parkar M, Andreou G, Brett PM, Ready D, Tonetti MS (2004). Periodontitis and atherogenesis: Causal association or simple coincidence. J Clin Periodontol.

[3] Saini R, Marawar PP, Shete S, Saini S, Mani A (2009). Dental expression and role in palliative treatment. Indian J Palliat Care.

[4] Scannapieco FA, Papandonatos GD, Dunford RG (1998). Associations between oral conditions and respiratory disease in a national sample survey population. Ann Periodontol.

[5] Scannapieco FA (1999). Role of oral bacteria in respiratory infection. J Periodontol.

[6] Mojon P (2002). Oral health and respiratory infection. J Can Dent Assoc.

